# Surgery

**Published:** 1895-03

**Authors:** W. M. Barclay


### SURGERY.

Of the carcinomata none are more important, from their common occurrence and the distress accompanying them, than cancer of the breast. While able and patient investigators in all countries are seeking the real cause of their origin, in the hope of throwing new light on the means of successfully treating them, their labours have not as yet enabled them to do so, and surgeons are left face to face with the old problem—how best to secure a complete removal of the primary tumour at an early date: for the controversy of the local and the constitutional origin of carcinomata is more and more dying out in favour of the former's claims. The local origin of cancer means then for most surgeons, that what they have most earnestly to seek is that method of operation which promises the best chance of removing the whole primary growth (not only as it appears visibly, but in its many invisible offshoots), as being at present the only practicable method of ridding the patient of her disease. In a paper delivered before the Harveian Society of London,[2] Mr. Watson Cheyne referred to certain investigations which explain some of the causes of the failure in the method of operation which was in general practice some years ago. Their value lies in enlarging our interpretation of what is a complete extirpation of the primary growth, and, as closely bound up with this, our knowledge of the possible routes of dissemination. As long ago as 1889, Heidenhain[3] pointed out the importance of the pectoral fascia, demonstrating that it is often involved in the cancerous process, even when the overlying breast is not adherent to it, and strongly advised its complete dissection off the pectoral muscle in all cases. The lymphatics of the breast (according to Sappey) converge to the nipple, and, after leaving this, form a sub-areolar network from which three or four trunks pass to the axillary glands. But it has also been found that lymphatics pass from outlying lobules and the deeper surface of the breast to the lymphatic network which lies in the pectoral fascia and on the surface of muscle immediately subjacent; and, further, that lymphatics pass from the sternal edge of the breast

[1] *Brit. M. J.*, 1895, vol. i., p. 368. [2] *Brit. M. J.*, 1894, vol. i., p. 289.

[3] *Verhandl. d. deutsch. Gesellsch. f. Chir.*, xviii., pt. 2, 1889, p. 1, and *Arch. f. klin. Chir.*, xxxix., 1889, p. 97.

to the sternum (and thence probably to the mediastinal glands), and sometimes to the opposite breast. Again, the breast has been shown to extend into the surrounding fat much more largely than was supposed. From these considerations, and the teachings of experience, Mr. Cheyne deduces the following rules of operation: (1) To remove all the skin co-extensive with the prominent part of the breast, never dissecting it from off the tumour; (2) To remove the pectoral fascia co-extensive with the breast, by dissecting off a thin layer of the pectoralis major; and (3) To dissect (not pull away) all the fat and fascia leading from the breast to the axilla, and the whole of the fat and glands in the axilla. He says he has generally been able to stitch up these extensive wounds by undermining the skin and using button-sutures; but this is a matter of comparatively small moment. He gives short notes of a series of cases in which he has employed this method; but it will conduce to clearness if his results are compared with that of other surgeons later on.

The method above advocated is a decided advance on the rather vague practice described as excising the breast and clearing out the axilla. With the view of still further facilitating the access to the axilla, especially its apex, Mr. Arbuthnot Lane[1] advocates the removal of the whole of the pectoral muscles with the adjacent breast, and the careful dissection from the axillary vessels and nerves of every bit of areolar tissue with lymphatics and glands. He was led to this conclusion by finding, in a recurrent case, that he had left a thick cord of infiltrated lymphatics which lay between the two pectoral muscles and entered the axilla above the pectoralis minor. When necessary, he clears out the subclavian triangle by dividing the clavicle and afterwards wiring the fragments together.

Dealing with this subject two valuable papers by Dr. Halsted[2] and Dr. Meyer[3] have recently appeared. The aim of each is identical, and, in Dr. Meyer's words, is "to extirpate the breast, the contents of the axillary, and the sub- and infra-axillary region, and the pectoral muscles in one mass." Their procedures are based on the belief that, in the ordinary methods, the wound may become infected by the division of cancerous tissues or lymphatics, or the cancer-element be pressed into the lymphatics in the frequent handling of the tumour and so be disseminated. The removal of the pectoral muscles is said not to interfere with the after-utility of the arm—the anterior fibres of the deltoid producing adduction;—but were it otherwise, it would be of no importance in such cases.

Dr. Meyer's operation is thus performed: Make the usual elliptical incision liberally, and prolong it at once well into the axilla. An incision is then carried from the junction of the middle and outer thirds of the clavicle to join the axillary extension at right angles. Three skin-flaps are reflected with as little of the

[1] *Tr. Clin. Soc.*, vol. xxvi., 1893, p. 85. [2] *Ann. Surg.*, vol. xx., 1894, p. 497.
[3] *Med. Rec.*, vol. xlvi., 1894, p. 746.

subcutaneous fat as is compatible with their vitality. Taking as landmarks the tendon of insertion of the pectoralis major, the cephalic vein, the clavicle, the edge of the latissimus dorsi, the sternal extremity of the clavicle, and the sternum, he begins by dividing the pectoralis major on the humerus, and its clavicular origin; dissects off the fat over the main vessels and nerves, freeing it from the bicipital sulcus till the edge of the latissimus dorsi is reached; after freeing it from the latter, the whole mass is reflected inwards on the subscapularis and teres major till the chest-wall is reached; the pectoralis minor is detached at its coracoid attachment; and finally the whole mass is severed at the costal origin of the pectoralis major.

The steps of Dr. Halsted's operation are as follows: (1) A liberal skin-incision is made surrounding the breast, and carried at once and everywhere through the fat down to the muscle; and a triangular flap of skin is dissected off the fat as far as the lower edge of the pectoralis major; (2) The costal origin of the pectoralis major is severed, and the muscle split, usually between its costal and clavicular portions, to a point opposite the scalene tubercle; (3) The clavicular origin, with the skin overlying it, is cut through close up to the clavicle (thus exposing the apex of the axilla), and the loose tissue under it dissected away; (4) The splitting of the muscle is continued to the humerus, and the tendon of insertion cut through close to that bone; (5) The whole mass is then raised forcibly and stripped from the thorax close to the ribs and pectoralis minor: when the lower border of this muscle has been passed and exposed, it is divided; (6) The tissues—more or less rich in lymphatics, and often cancerous—which lie over the muscle at its coracoid attachment are divided as far out as possible, and reflected inwards, clearing this part of the muscle, which is then drawn upwards; and the small blood-vessels underneath it are carefully separated and cleaned, and tied close to the axillary vein; (7) The axillary and the beginning of the subclavian vein having been thus exposed as high as possible, the contents of the axilla are to be dissected away "with the sharpest possible knife" (not torn away), and not a particle of fat is to be included in ligatures applied to vessels; (8) The vessels thus cleaned, the axillary contents are rapidly stripped from the side of the thorax and the posterior wall of the axilla, and finally are severed at the line of attachment of the reflected skin-flap. The coracoid portion of the pectoralis minor is removed, as may be the central portion if thought advisable. Dr. Halsted further advises in almost all cases clearing out the subclavian triangle in the same way as the axillary space, since he found that in at least five out of his fifty cases the supra-clavicular glands were enlarged and cancerous. He, however, gives no details as to the method he adopts in carrying out this suggestion. Dr. Meyer,[1] referring to this point, concurs in the suggestion, and proposes prolonging

[1] *Loc. cit.*

his second incision over the clavicle. Dr. Halsted claims for his operation that it is almost bloodless, and that therefore shock is reduced to a minimum. Also, by including only one side of the triangular skin-flap in the suture, and using the rest of it "as a lining for the fornix of the axilla"—the flap being "held in place by a carefully applied dressing,"—he says he prevents gluing of the arm to the side. He does not drain the axilla, and says he invariably gets primary union.

In fourteen cases of mammary cancer in which I have operated, I have always systematically cleared out the axillary fat up to the extreme apex, and all round the axillary vessels; and down the posterior wall, exposing the subscapular vessels and nerve. In several instances I have divided both pectoral muscles, clearing these thoroughly of all fat, and gaining very complete access to every part of the axillary hollow. I have in each case united the muscles again with loose catgut sutures. I have for some time past made an opening for a drainage tube to be passed into the axilla at the dependent part of the lower skin-pouch left after excision, and sutured the operation-wound completely. In seven cases there was union by first intention throughout; in several more this occurred, except where the sides of the wound could not be brought together; and in a few cases a limited amount of suppuration occurred. I have had no serious trouble with any breast case.

In trying to estimate results by the special methods advocated, all the observers adopt the law formulated by Volkmann, that "when, after an operation, a whole year has elapsed without recurrence, one may hope for a permanent cure; after two years this cure is probable; at the end of three years it becomes almost certain."

Dr. Halsted has added at the end of his paper the statistics of a large number of foreign surgeons; but the test on which he seems to chiefly rely in comparing his results with theirs is that of local recurrence; and he further rather obscures the comparison by dividing local recurrences into what he calls truly "local"—*i.e.*, recurrence in the scar itself, using this word in its widest sense,—and "regional"—*i.e.*, skin metastases occurring at a greater or less distance from the line of incision. In this limited sense of "local" his recurrences are remarkably few, only six per cent.; but if the "local" and "regionary" recurrences are combined, the percentage is twenty-seven. A more satisfactory way is to compare the numbers of patients that survive the operation two years or more without recurrence in any series of cases. Of eighteen cases of Dr. Halsted's, nine, or fifty per cent., remained well, of whom four had survived three or more years. Mr. Watson Cheyne quotes fifteen cases operated upon more than two years previous to his paper. Of these, ten, or 66·6 per cent., remained well, of whom three had survived three years or more. Of seven cases in which I operated more than two years ago, one is living and free from recurrence three and a half years, and another two years, after operation. A third case, operated upon three years and five months ago, I have not been able to trace. Four died at periods varying from one and a half years to four months after treatment.

Dr. Lewis S. Pilcher[1] made a study of such cases (among others) in the Methodist Episcopal Hospital, Brooklyn. A "radical" operation was performed, *i.e.*, systematic removal of axillary fat and glands; wide skin-incision; and, in many cases, excision of large portions of the pectoral muscles. Four out of twenty-four cases had survived three years and over. The general estimate of permanent recurrences is given by Mr. Cheyne[2] as ten or fifteen per cent.

Unfortunately the three-year rule of Volkmann is not a rigid one; and several instances are given by Dr. Halsted and others where patients have suffered a recurrence three or four years after operation, locally as well as internally. Any measures, however, which enable us more frequently to insure immunity from local recurrence are a real advance. To increase more and more the prospects of conferring final immunity by an adequate operation, it will probably become only a question of getting patients to consent to the removal of this disease in its earlier stages.

In this connection a paper by Dr. W. B. Coley, of New York,[3] is of the greatest interest. For although the treatment here reported for inoperable malignant tumours seems especially efficacious in the class of sarcomata, yet there seems some hope that the results in carcinomata may improve. The treatment is based on the well-known fact that erysipelas attacking malignant growths has sometimes resulted in their cure. The method consists in injecting locally into the tumour, at intervals of from twenty-four to forty-eight hours, or longer, according to the reaction produced, the mixed toxins of the streptococcus of erysipelas and the bacillus prodigiosus, the cultures being sterilised at a low temperature, but not filtered. After cooling, some thymol is added as a preservative. Of this preparation the dose is one to eight minims. It is of rather uncertain strength; and Dr. Coley advises always beginning with the lowest dose, and gradually increasing it until the desired reaction is obtained, viz., a rise of temperature to 103° or 104° F. This reaction is only temporary, passing off in a few hours. The cases treated have all been either growths which had repeatedly recurred and were finally inoperable, or such as from the first were not fit for operation. In many cases the tumour disappeared by a process of necrosis and disintegration, but in some by absorption. One very remarkable case of the latter kind is quoted in which an enormous spindle-celled

[1] *Ann. Surg.*, vol. xx., 1894, p. 1. [2] *Loc. cit*
[3] *Med. Rec.*, vol. xlvii., 1895, p. 65.

sarcoma, involving nearly the whole of one side of the chest, back and front, entirely disappeared in four months.

He had treated (by the combined toxins, filtered, as he originally prepared them) up to May, 1894, twenty-five cases of sarcoma, eight of carcinoma, and three of carcinoma or sarcoma; and since that date (by the combined toxins, unfiltered, as described above), thirteen sarcomata and eleven carcinomata. In the cases of sarcoma only, can Dr. Coley claim any cures; and of the combined thirty-eight cases, he thinks nine promise to be permanently successful: but, although none of the carcinomatous tumours disappeared, the injections seemed to have an undoubted retarding influence, in some cases to an extraordinary degree.

The method is, of course, still on its trial; but enough evidence has been adduced to more than justify an extended and careful employment of it in these otherwise hopeless cases. The discomfort and risks seem very slight, while the issues at stake are enormous.

W. M. Barclay.

